# Wavelengths and irradiances modulate the circadian rhythm of *Neurospora crassa*

**DOI:** 10.1371/journal.pone.0266266

**Published:** 2022-03-30

**Authors:** Peijun Wen, Fuyun Tan, Menglai Lei, Muhammad Saddique Akbar Khan, Weihua Chen, Xiaodong Hu

**Affiliations:** State Key Laboratory for Artificial Microstructure and Mesoscopic Physics, School of Physics, Peking University, Beijing, China; Karlsruhe Institute of Technology, GERMANY

## Abstract

The circadian rhythm affects the biological evolution and operating mechanisms of organisms. The impact of light on the circadian rhythm is a significant concern for both biology and human well-being. However, the relation between different wavelengths, irradiances, and circadian rhythm is unknown. In this study, we compared the effects of four different monochromatic light-emitting diode (LED) light and two different irradiances on the circadian rhythm of a wild-type *Neurospora crassa*. The results demonstrated that the circadian rhythm of *Neurospora crassa* can be modulated by violet (λp = 393 nm), blue (λp = 462 nm), and green (λp = 521 nm) light, regardless of the irradiances, in the visible region. Unexpectedly, for the yellow light (λp = 591 nm), the 2 W/m^2^ light had a more significant impact on circadian rhythm modulation than the 0.04 W/m^2^ light had. Considering the highest energy of yellow light (2.25 eV) is lower than the High Occupied Molecular Orbital (HOMO)-Lowest Unoccupied Molecular Orbital (LUMO) gap of WC-1 (2.43 eV). We speculate that there may be other potential photoreceptors that are involved in circadian rhythm modulation. The HOMO-LOMO gaps of these proteins are greater than 1.98 eV and less than 2.25 eV. These results provide a strong foundation for a deeper understanding of the impact of different light on the circadian rhythm and also shed light on the identification of new circadian rhythm modulation photoreceptors.

## Introduction

The circadian rhythm has been shown to be present in humans, animals, plants, and some microorganisms [1–6] which is the result of adaption to geosynchronous. The circadian rhythm affects the biological evolution and operating mechanisms of organisms [7, 8]. The system of the internal circadian clock is formed by three parts that are the input, the central oscillator, and the output, of these three the central oscillator is the most important. Generally, the oscillator of circadian rhythm is considered to be influenced by two primary inputs that are the light and temperature, respectively [9]. The impact of light and temperature is imposed upon the internal rhythm through the oscillator which operates at the molecular level. The internal rhythm that is generated by the oscillator is thereafter through the output pathway.

Model organisms from the fungi, botany, insect and animal groups are usually used in the study of the circadian rhythm [10]. Although those model organisms are evolutionarily different, their oscillators are all based on a transcriptional-translational feedback loop (TTFL) [11–14]. *Neurospora crassa* is a reliable and suitable organism [15, 16] to be used in circadian rhythm studies because its genetics can be easily manipulated and its conidiation, which is usually regarded as a signal of its circadian rhythm, is easily observed [17–20]. The study of light regulation in *Neurospora* has been becoming one of the important research topics. Corrochano et al. have reported outstanding work on light regulation in *Neurospora*. They had done the mutational analyses of photoadaptation [21], and the activation of conidiation by the blue light [22]. Besides, blue light has been proved to enhance conidiation and other asexual development processes, such as entrainment of the conidiation circadian rhythm [23, 24]. Wang et al. showed that light and oxidative stress regulated the switch via light-responsive and ROS pathways in *Neurospora crassa* [25]. Bieszke et al. indicated that the green light receptor probably regulated the conidial developmental processes as an adjunct to the blue light signaling pathway [26]. Both these two studies [25, 26] described the existence of phytochromes and opsins in the fungus. Those previous researches provide a nice point of view to discover the effects of light on *Neurospora crassa*. As we can see the topic of light regulation in *Neurospora* gains a lot of attention.

In this study, we focus on the relation between light and the circadian rhythm of *Neurospora crassa*. The circadian rhythm of *Neurospora crassa* is mainly influenced by light and temperature [9], which are two kinds of energy. However, the effect of temperature on the circadian rhythm of *Neurospora crassa* is more complicated than that of light because the temperature can have both physical and chemical impacts on the oscillator, such as influencing the activity of the enzyme [27, 28]. On the other hand, the irradiance of the light field is easier to quantify than the heat transferred by the temperature field to the sample. Thus, the effect of light on *Neurospora crassa* can be quantified more accurately and studied more precisely than the effect of temperature. The previous studies on the relation between light and the circadian rhythm of *Neurospora crassa* paid more attention to other variables and effects instead of the nature of the light sources, such as the spectrum, illuminance, intensity.

The development of semiconductor optoelectronics [29] has led to the invention of more advanced and varied forms of lighting. The light-emitting diode (LED) is a very good example because LED is environmentally friendly, easy to control its parameters, besides, LED has long-life use and provides a good quality of light [1, 30]. More importantly, LED has a narrow full width at half maximum (FWHM) and produces less heat [31]. Thus, LED is an ideal illumination source for accurate investigation on the effects of light on *Neurospora crassa*. In this study, we explore the relation between wavelength, irradiance, and circadian rhythm of *Neurospora crassa*.

**Fig 1:**
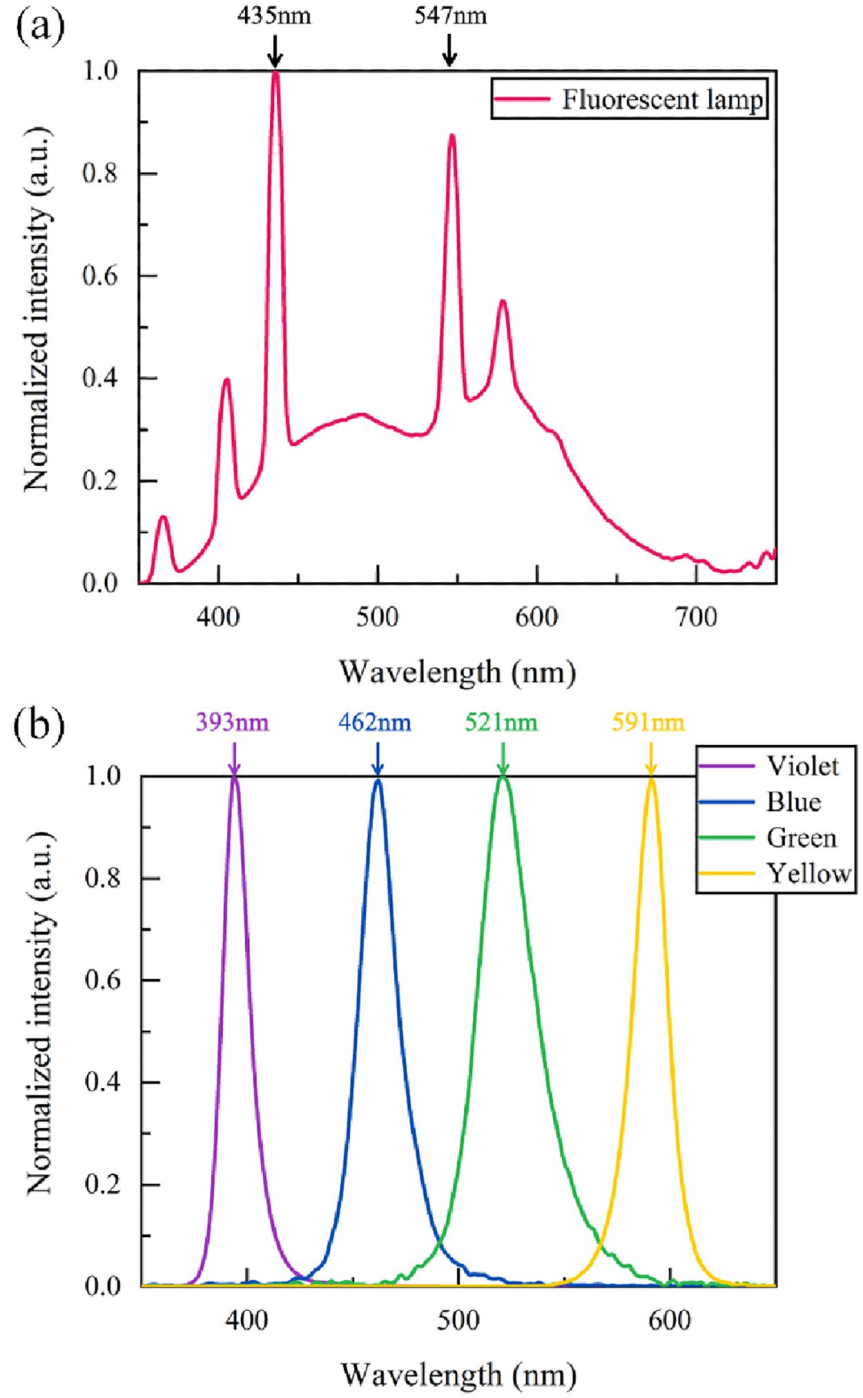
The spectra of the fluorescent lamp and LEDs. (a) The spectrum of the fluorescent lamp. (b) The spectrum of the LEDs. Left to right: violet (FWHM = 15.7 nm), blue (FWHM = 23.7 nm), green (FWHM = 33.9 nm) and yellow (FWHM = 20.5 nm) LEDs. We replaced the fluorescent lamp with the monochromatic LED for the light intervention experiment.

The lighting inside biological incubators is usually fluorescent lamps. Fluorescent lamps create visible light by using ultraviolet light to activate the fluorescent powder coated inside the glass tube and it provides a fairly diverse light spectrum (Fig 1a). Therefore, it is hard to investigate the effects of different wavelengths influencing the circadian rhythm of *Neurospora crassa* by using traditional fluorescent lamps. In this study, we replaced fluorescent lamps with monochromatic LED light (S1 File) with narrow FWHM, which means the specific relation between different wavelengths of light and the circadian rhythm of *Neurospora crassa* can be researched precisely. Furthermore, LED produces less heat during the experiment so that the thermal disturbance is minimized. Moreover, the stability of LED can be used for long-term intervention experiments.

## Materials and methods

This study employed four monochromatic LEDs with different wavelengths: monochromatic violet, blue, green and yellow (Fig 1b). The effects of different spectral irradiance on *Neurospora crassa* were also examined. The four LEDs were adjusted at two different irradiance levels for light interventions, which were 2 W/m^2^ and 0.04 W/m^2^. The spectrum and irradiance were accurately measured by the illuminometer at the Race Tube position of *Neurospora crassa*.

The *ras-1*^*bd*^ of *Neurospora crassa* [32] was selected as the sample in this study because of its unique advantages. For example, the *ras-1*^*bd*^ can grow on the basic culture medium without other special conditions, and the conidiation of *Neurospora crassa* can be easily observed and tested during its growth process. Moreover, the *ras-1*^*bd*^ can reflect the conidiation development of the *Neurospora crassa* without other genetic influences [33]. These characteristics make *ras-1*^*bd*^ an ideal choice for this research.

We intended to test whether the sample can be affected by different wavelengths and be synchronized from the natural circadian rhythm of the sample, which is approximately 22 hours per period [34], to 24 hours per period cycle. The LED lighting installed in the incubator was set to 12 hours on and 12 hours off schedule. In order to regulate the lighting time precisely, an Arduino single chip was used to control the LEDs. In this study, there were two experimental groups and one control group (Table 1). Both experimental groups were subjected to illumination by violet, blue, green, yellow monochromatic LEDs (only one monochromatic light per experiment) with the spectral irradiance being set to 2 W/m^2^ and 0.04 W/m^2^ respectively, because 2 W/m^2^ and 0.04 W/m^2^ of LEDs were the maximum and minimum irradiance due to the space and distance inside of the incubator. The control group was left in total darkness.

**Table 1:** Experimental parameters.

	Number of replicates	Spectral irradiance	Light conditions	Temperature	Periods
**Experimental group 1**	N = 24	2 W/m^2^	12 h on / 12 h off	25°C	6 days
**Experimental group 2**	N = 24	0.04 W/m^2^	12 h on / 12 h off	25°C	6 days
**Control group**	N = 24	N/A	Total darkness	25°C	6 days

In order to control the variable, all the samples were put inside the incubators and maintained at a consistent temperature of 25°C. The experiment started from shorter wavelength light to longer wavelength light (violet, blue, green, yellow light). The samples of *Neurospora crassa* were placed in the starting point of the Race Tubes [35]. The samples grew from these starting points to the end of the Race Tubes over the course of the experiments. Each monochromatic light experiment had 6 independent Race Tubes, the growth lengths were observed and marked by black spots every 12 hours. Meanwhile, after the experiment, the samples in the Race Tubes were scanned to obtain the image. Based on a method of recording and analysis of rhythms from the previous work [36], we developed our Python program. After getting the scanning photo of the Race Tubes, we put it into a Python program. Firstly, we turned this RGB figure into a grey figure. Then we used a certain piece of the program to recognize the black spots which we marked during the experiment. Once we got the coordinates of every black spot, the growth front of mycelium and the place where the conidiation is densest in the image can be analyzed. We used the picture-process method in our Python program to obtain the density-curve of conidiation. Then, filtering, smoothing, and finding peaks processes were done for these curves by using relative functions in the *scipy*.*signal* toolkit. The peak of the curve represented the maximum of conidiation, and the peak array reflected the periods of conidiation. Thus, the circadian rhythm of *Neurospora crassa* can be calculated. After that, we used SPSS (IBM Corporation, USA) for data analysis. The experimental group 1, experimental group 2 and control group had 24 Race Tubes respectively. The independent-sample t-test is used to analyze the difference between the experimental group and the control group. The p-value less than 0.05 was considered significant.

## Results and discussions

**Fig 2:**
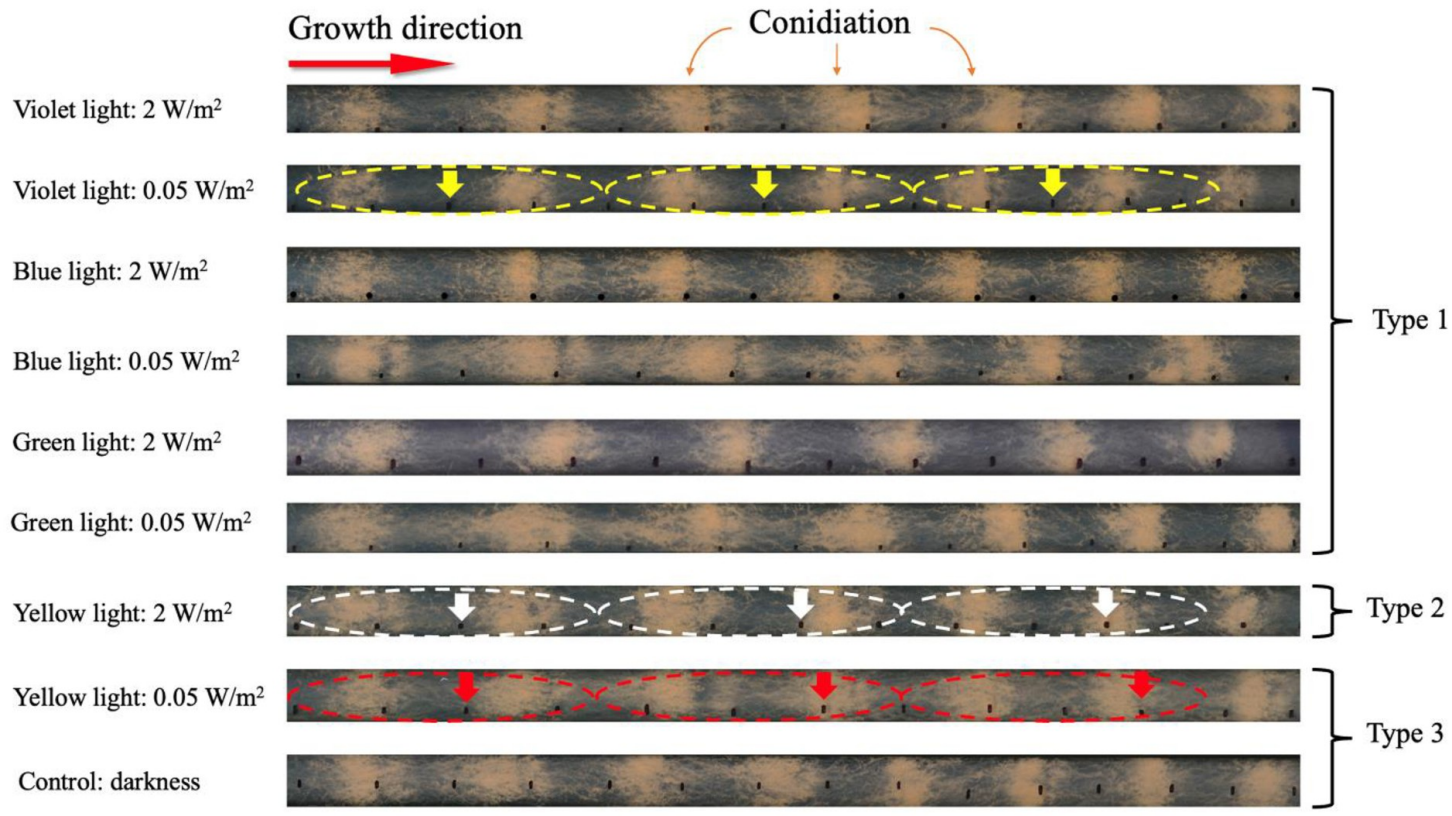
Growth results of the samples. The samples were placed on the starting points on the far-left side of the Race Tube. It grew up from the left side to the right side to the end of the Race Tube. The orange sections in the picture mark the conidiations. The black spots on the Race Tubes were the growth length of the sample, which were marked every 12 hours. There were three different types of growth results, and the arrows indicated a black spot at 24 hours, 72 hours and 120 hours. Type 1 (yellow arrows) showed that a 24 hours period of circadian rhythm, the three arrows were always in the middle of conidiation. Type 2 (white arrows) indicated that a 22.3 hours per period, the second arrow approached conidiation, and the third arrow was included in conidiation. Type 3 (red arrows) was around 21.7 hours per period, the second arrow was included in conidiation, the third arrow exceeded conidiation.

After the light intervention, there were three different types of growth results that represented the circadian rhythm period of 24 hours, 22.3 hours, 21.7 hours, respectively (Fig 2).

**Fig 3:**
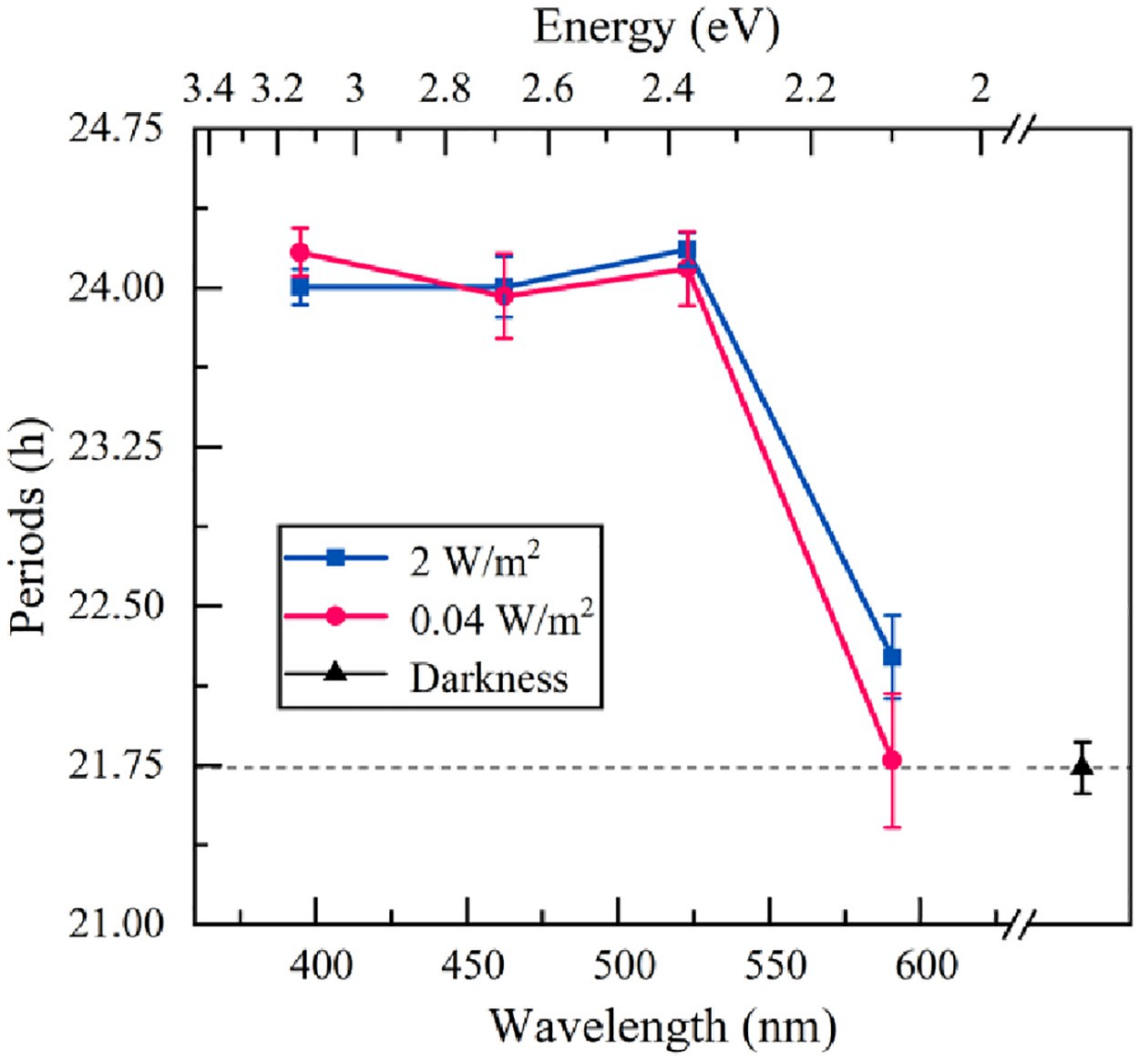
The results of circadian rhythm periods (Mean ± SEM). The violet, blue, and green light can modulate the circadian rhythm of the sample from 21.7 hours to around 24 hours, but that yellow light weakens the modulating effect. Both the circular and square points from the left to right represent the violet, blue, green and yellow light. The black triangle is the control group.

The results illustrated (Table 2 and Fig 3) that the original circadian rhythm of the sample was 21.7 hours (control group). Following the light intervention, the results of the experimental groups showed that the violet, blue, and green light with irradiances of 2 W/m^2^ and 0.04 W/m^2^ were able to modulate the circadian rhythm of the sample from 21.7 hours to 24 hours successfully. On the other hand, neither 2 W/m^2^ nor 0.04 W/m^2^ of yellow light could reprogram the circadian rhythm of the sample from 21.7 hours to 24 hours (Fig 3). However, the circadian rhythm did change to 22.3 hours under 2 W/m^2^ yellow light which was found to be statistically significant compared to the control group (p<0.001), and there were no remarkable differences between the circadian rhythm (21.8 hours) of the sample under 0.04 W/m^2^ yellow light and the control group (p = 0.421).

**Table 2:** The circadian rhythm (hours±SEM) and the statistical results compared with the control group.

	Irradiance: 2 W/m^2^				Irradiance: 0.04 W/m^2^				Control
	Violet	Blue	Green	Yellow	Violet	Blue	Green	Yellow	Darkness
**Average**	24.0 ±0.1	24.0 ±0.1	24.2 ±0.1	22.3 ±0.2	24.2 ±0.1	24.0 ±0.2	24.1 ±0.2	21.8 ±0.3	21.7 ±0.1
**p-value**	<0.001	<0.001	<0.001	<0.001	<0.001	<0.001	<0.001	0.421	

In this study, the circadian rhythm of the sample was reset from 21.7 hours to around 24 hours by the violet (λp = 393 nm), blue (λp = 462 nm), and green (λp = 521 nm) light LEDs which were switched between light and dark mode on a 12-hour schedule. These results were in close agreement with previously published work of circadian clock of wild type. There were some biological responses from around 400 nm to 500 nm [37]. This interruption to the light input signal caused the inner biological clock of the sample to synchronize with the external environment. *Neurospora crassa* has two kinds of LOV (light, oxygen, or voltage) domains of the blue-light photoreceptors. These are the White Collar-1 (WC-1) and Vivid (VVD), respectively [38]. WC-1 is the main photoreceptor in *Neurospora crassa*, and is an important protein that affects circadian rhythm. The WC-1 can be combined with flavin adenine dinucleotide (FAD) to respond to light signaling [37]. The peak value of the FAD absorption spectrum is 450 nm, which indicates that WC-1 is the most sensitive to blue light, the cut-off wavelength of light absorption of FAD is around 510 nm [39, 40]. Thus, the gap between the Highest Occupied Molecular Orbital and the Lowest Unoccupied Molecular Orbital (HOMO-LUMO gap) of FAD is about 2.43 eV.

According to the spectrum of LEDs in Fig 1b. The energy ranges corresponding to the green light (λp = 521 nm) and yellow light (λp = 591 nm) are 2.07–2.70 eV and 1.91–2.25 eV, respectively. The highest energy of green light (2.70 eV) is higher than 2.43 eV (WC-1), so the green light can be absorbed by WC-1. On the other hand, the highest energy of yellow light (2.25eV) is lower than the HOMO-LUMO gap of WC-1, so the WC-1 definitely cannot absorb yellow light, however, the circadian rhythm results showed that yellow light with 2 W/m^2^ can induce the circadian periods of sample to be slightly longer than their original periods (p<0.001). Therefore, in addition to WC-1, we speculate that there are other proteins that are also involved in the modulation of the circadian rhythm of the sample. Although these proteins cannot affect the circadian rhythm as much as WC-1, they can partly modulate circadian rhythm, and absorb yellow light. According to the energy range (1.91 eV-2.25 eV) of yellow light, we consider that the HOMO-LUMO gap of the potential proteins is lower than 2.25 eV, which makes it possible to absorb the yellow light.

Furthermore, according to the yellow light results, the yellow light with 2 W/m^2^ can induce the circadian periods of the sample, while the yellow light with 0.04 W/m^2^ cannot change the circadian rhythm at all. This illustrated that the irradiance was not the key factor to affect the circadian rhythm of *Neurospora crassa* when the energy was high enough (violet light, blue light and green light), however, the irradiance was important to influence the circadian rhythm when the energy was low (yellow light). The irradiance-dependence of the effect of the yellow-light intervention could be explained by the poor but non-negligible overlap between the absorption spectrum of the potential proteins and the emission spectrum of the yellow LED, which results in a relatively low electron transition probability from the HOMO level to the LUMO level. Therefore, an irradiance of sufficient magnitude is required to affect the circadian rhythm of *Neurospora crassa* in the yellow light case.

## Conclusion

We investigate the relation between the wavelength and circadian rhythm of *Neurospora crassa*. The effective wavelength range that can modulate the circadian rhythm of *Neurospora crassa* is from violet to green light. In this range, the circadian rhythm of *Neurospora crassa* is only related to the wavelength of light, and independent of the irradiance of light (both 2 W/m^2^ and 0.04 W/m^2^). Furthermore, although yellow light cannot modulate the circadian rhythm of *Neurospora crassa* as effective as violet light, blue light, and green light, 2 W/m^2^ yellow light can also affect the circadian rhythm of *Neurospora crassa*. Thus, we speculate that there are other proteins (in addition to WC-1) that must be present to modulate the circadian rhythm based on the calculation between the HOMO-LUMO gap of WC-1 and yellow light energy. This study has found a significant variation between the wavelength of light and the circadian rhythm of *Neurospora crassa*. These results will provide a strong foundation for a deeper mechanism study.

## Supporting information

S1 File This file contains all of the spectra data of the fluorescent lamp and LEDs. (XLSX)
